# Real-Time Closed-Loop Feedback in Behavioral Time Scales Using DeepLabCut

**DOI:** 10.1523/ENEURO.0415-20.2021

**Published:** 2021-04-13

**Authors:** Keisuke Sehara, Paul Zimmer-Harwood, Matthew E. Larkum, Robert N. S. Sachdev

**Affiliations:** 1 Institute of Biology, Humboldt University of Berlin, Berlin D-10117, Germany; 2 Department of Physiology, Anatomy and Genetics, University of Oxford, Oxford OX1 3PT, United Kingdom

**Keywords:** behavioral tracking, closed-loop systems, deep-neural network

## Abstract

Computer vision approaches have made significant inroads into offline tracking of behavior and estimating animal poses. In particular, because of their versatility, deep-learning approaches have been gaining attention in behavioral tracking without any markers. Here, we developed an approach using DeepLabCut for real-time estimation of movement. We trained a deep-neural network (DNN) offline with high-speed video data of a mouse whisking, then transferred the trained network to work with the same mouse, whisking in real-time. With this approach, we tracked the tips of three whiskers in an arc and converted positions into a TTL output within behavioral time scales, i.e., 10.5 ms. With this approach, it is possible to trigger output based on movement of individual whiskers, or on the distance between adjacent whiskers. Flexible closed-loop systems like the one we have deployed here can complement optogenetic approaches and can be used to directly manipulate the relationship between movement and neural activity.

## Visual Abstract

**Figure:**
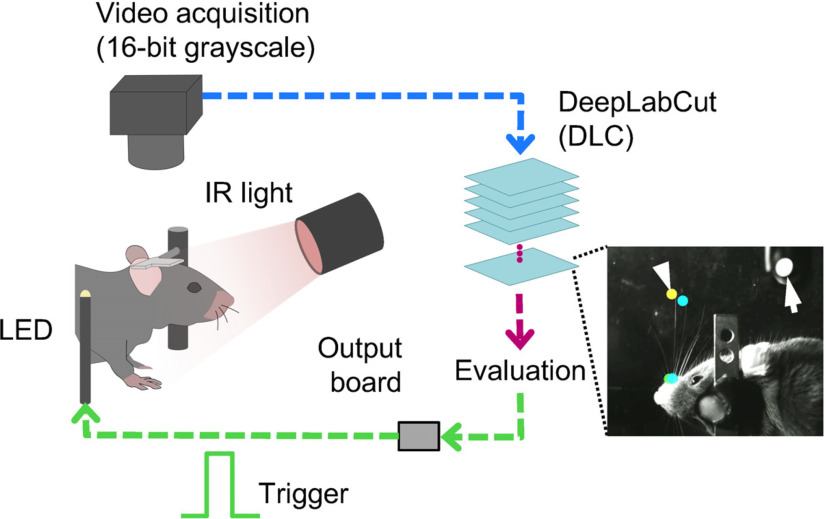

## Significance Statement

Here, we deploy a deep-neural network (DNN)-based fast feedback method that can be used to reconfigure feedback to mice based on the movement of particular whiskers, or on the distance between particular whiskers. Our system generates feedback within 10.5 ms. Methods like the one we present here will steadily become part of the standard toolset for manipulating the interaction between the animal and its environment in behavioral time scales.

## Introduction

Behavior is a sequence of motor actions controlled and monitored by a pattern of neural activity that is distributed throughout the brain. In most contexts, neural activity related to movement can be detected in a variety of cortical, subcortical, brainstem and spinal circuits. Our toolset for directly manipulating and monitoring neural activity is vast and sophisticated. By comparison, the tools we have for manipulating and monitoring behavior are somewhat meager. Understanding the causal relationship between neural activity and decision-making or movement requires tools that work flexibly and rapidly.

High speed videography is a standard tool for monitoring behavior and relating behavior *post hoc* to neural activity. Traditionally, video data have been analyzed manually. More recently, various algorithms have been developed for automating movement detection (Knutsen et al., 2005; Voigts et al., 2008; Perkon et al., 2011; Clack et al., 2012; Ohayon et al., 2013; Giovannucci et al., 2018; Dominiak et al., 2019; Vanzella et al., 2019; Betting et al., 2020; Petersen et al., 2020). With the development of DeepLabCut, a marker-less pose-estimation toolkit based on deep learning (Mathis et al., 2018), computer vision approaches are being used for monitoring poses of animals and for tracking the movement of virtually any part of the body. The main advantage of DeepLabCut over other algorithms is that it can be deployed on any and all parts of the body that are imaged in videos. Furthermore, DeepLabCut is easy to use, and it is easy to train with additional datasets.

These standard approaches for movement tracking have been supplemented or complemented with real-time monitoring and manipulation of behavior (Nashaat et al., 2017; Cao et al., 2018; Sehara et al., 2019). One uses low resolution, inexpensive, color tracking cameras for monitoring the movement and position of animals at a latency of ∼30 ms (Nashaat et al., 2017). A more recent approach used machine learning algorithms that work at a latency of ∼100 ms or less (Cao et al., 2018). Yet another uses neuromorphic cameras and has a much shorter latency (2 ms) for tracking whiskers (Sehara et al., 2019). Here, we describe a real-time DeepLabCut implementation that can be used to track movement at a resolution of 10–15 ms and generate triggers in real time at a latency of 10.5 ms. We performed our proof-of-principle experiments in the rodent whisker system, a complex sensorimotor system which requires tracking of similarly shaped tactile sensors, each moving at 20–25 Hz. We expect our system to be applicable to real-time tracking of movement of any set of body parts in almost any behavioral application.

## Materials and Methods

### Animal experiments

All procedures using mice were performed in accordance with protocols approved by the Charité–Universitätsmedizin Berlin and the Berlin Landesamt für Gesundheit und Soziales (LaGeSo) for the care and use of laboratory animals.

#### Animals

Six male C57BL/6 mice (RRID:IMSR_JAX:000664) housed under a 12/12 h reverse light/dark cycle were used in this study.

#### Surgery

Animals were anesthetized with ketamine/xylazine (90/10 mg/kg body weight) and placed on a feedback-regulated heating pad. After subcutaneous lidocaine injection, skin and fascia were removed from the skull. A lightweight aluminum headpost was attached to the skull using a Rely-X (3 M) cement, followed in some cases by Jet acrylic black cement (Dominiak et al., 2019; Sehara et al., 2019). Animals were monitored during recovery and were given antibiotics (enrofloxacin) and analgesics (buprenorphine and carprofen). After a recovery period, the animals were habituated to the experimenter’s handling and to being head-fixed at the setup.

#### Animal imaging setup

**Figure 1.**
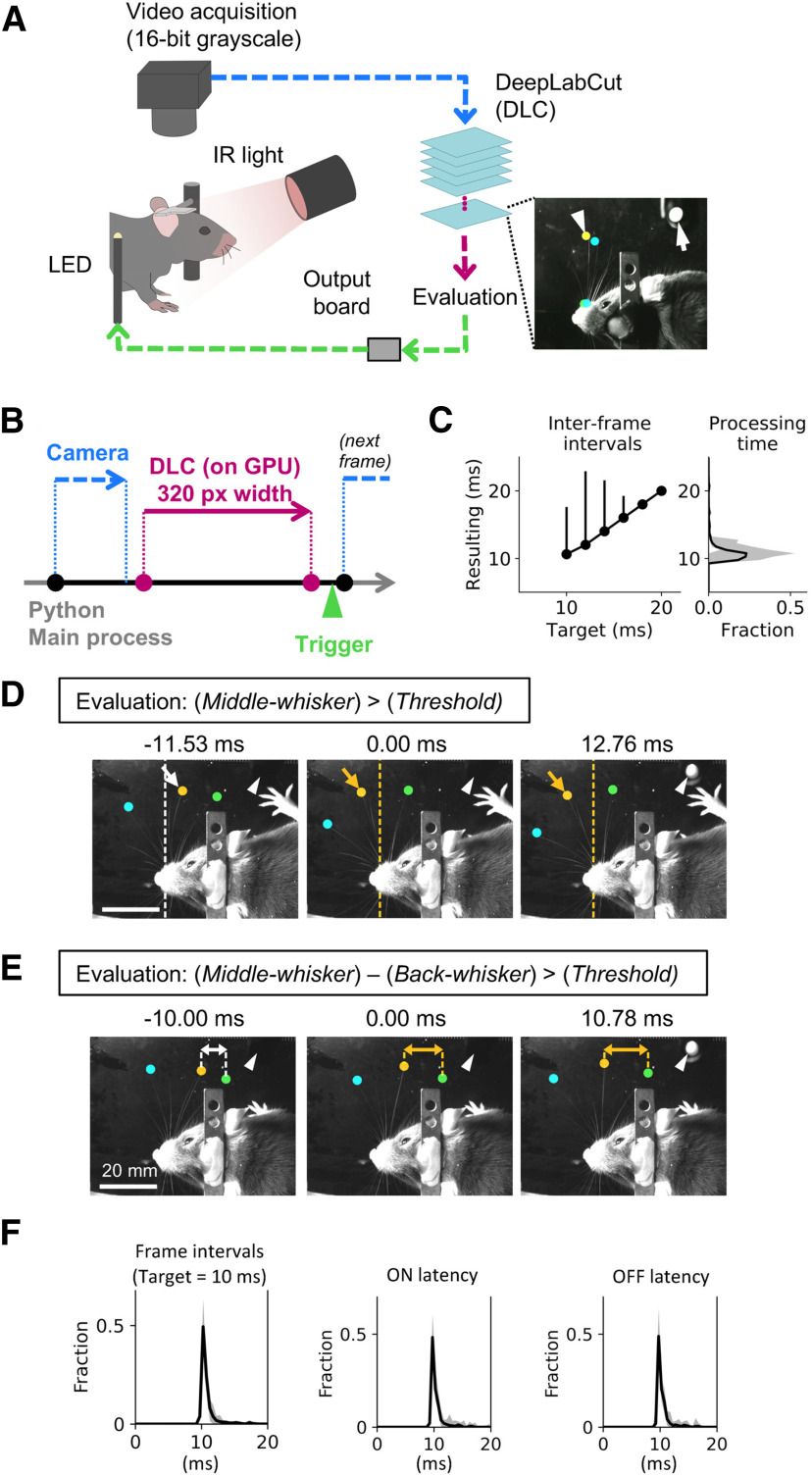
Proof of concept of real-time feedback based on whisker position. ***A***, Schematic of the setup. The mouse was head-fixed under the camera, and high-speed video was acquired under infrared (IR) illumination. Whisker positions were estimated from each frame. Digital output, turning on an LED, was generated based on estimated positions using DeepLabCut. The inset shows the example annotations based on the output. Estimated positions were stored after acquisition and used for *post hoc* annotation. Arrowhead shows the estimated position of a whisker tip. Arrow points to the flashing LED. ***B***, Flow of acquisition and trigger output. Acquisition of a frame (blue) starts with a trigger being generated by the busy-wait algorithm. The acquired frame was passed on to DeepLabCut-mediated body-part estimation (red) after subsampling the frame to half the original size. The status of trigger output was determined based on the estimated body-part positions and was generated as TTL signal (green). ***C***, Acquisition speed. The effect of changing the busy-wait timer settings (*x*-axis) on the interframe acquisition intervals (median and 5% confidence intervals of *N* = 3000–3500 frames per setting). The right panel shows the average histogram for the total processing time per frame (median and best/worst cases out of *N* = 6 sessions). Exposure of 400 μs was used, and frames were subsampled to 320 pixels in width before being processed. ***D***, ***E***, Example consecutive frames in a single representative session. The positions of three whiskers (cyan, orange, green) on one side of the mouse’s face were estimated. Flashes of the LED in the field of view (arrowheads) reported the generation of output triggers in real time. Videos in ***D***, ***E*** differ in the way the estimated whisker positions were evaluated during the session. In ***D***, the trigger (arrowhead) was generated when the middle whisker (orange, arrows) protracted across the arbitrary border (dotted lines). The color of the border and the arrow indicates the status of trigger output (white: off, orange: on). ***E***, The trigger was generated when the horizontal distance (arrows) between the two whiskers on the back (orange, green) went above the arbitrary threshold. The color of the arrow indicates the status of trigger output (white: off, orange: on). Scale bars: 20 mm. ***F***, Latency profile during real-time acquisition and trigger generation (median and best/worst cases out of *N* = 26 videos from six behavioral sessions taken from three animals). When the interframe intervals were targeted at 10 ms the resulting frame intervals (left), the trigger on-event latency (center) and the trigger off-event latency were stable at ∼10 ms.

The mouse was head-fixed under infrared LED illumination (Fig. 1*A*). A 16-bit grayscale camera (DMK 37BUX287, Imaging Source) captured high-speed videos of mouse behavior from the top. For acquisition, a lens with 50-mm focal length was used at f/2.8. The exposure was set at 100–400 μs. The acquired frames were transferred via USB3.1 (Gen.1) communication to the host computer. To capture trigger outputs from the real-time tracking program, a 3-mm LED was located on the side of the animal, in the field of view of the camera. Each imaging session lasted for <15 min.

### Imaging system

The host computer (3.6 GHz Intel Core i7-9700K, 64-GB RAM) ran Ubuntu 18.04, and was equipped with the NVIDIA GeForce RTX 2080 Ti graphics card (11-GB RAM). We built a Python-based custom program (the “Pose-Trigger” library), along with a thin wrapper python library for DeepLabCut-based real-time tracking (the “dlclib” library) to run image acquisition and feedback generation (Fig. 1*A*,*B*). It runs in loops of image acquisition, position estimation, evaluation of positions, and feedback trigger generation, optionally with storage of acquired images and estimated positions into the disk. We designed the program so that each step after image acquisition can be separately turned on and off. To ensure that we achieve the shortest interframe intervals, and to keep frames from dropping, software triggers based on the “busy wait” algorithm were generated within the script and were used to capture each frame (Fig. 1*C*).

#### Image acquisition

Acquired frames had dimensions of 640 pixels (width) and 480 pixels (height). We built a custom Cython (RRID:SCR_008466)-based library (“timedcapture”) for managing image acquisition on top of the Video4Linux2 (V4L2) layer. Although the library was optimized for the use with our camera hardware, any V4L2-compliant camera can be used. To reduce acquisition latency on the host computer, the frame buffer size was set to one. To detect the timing of frame capture events, the “strobe-trigger” digital output of the camera was turned on during acquisition.

#### Position estimation

DeepLabCut 2.1.3 (Nath et al., 2019), with CUDA Toolkit 10.1 and Tensorflow 1.13.1, was used to perform marker-less position estimation. The “batch size” of the deep-neural network (DNN) model was set to one, i.e., it was designed to process one frame at a time. On top of the trained DNN model, we added the “GPU-based inference stage” as it was introduced in DeepLabCut 2.1 (Nath et al., 2019). The incoming image was transformed into 8-bit grayscale. Before being fed to the DNN model, the image size was down-sampled to 320 pixels in width (i.e., half the original size), unless otherwise specified, using Python bindings of the OpenCV library (RRID:SCR_015526; opencv-python, version 3.4.9.33; https://opencv.org/).

#### Position evaluation

The experimenter could interactively enter the Boolean expression to be evaluated while the program was running (Fig. 1*D*,*E*). Thus, theoretically, any evaluation algorithm can be implemented and changed without re-installing or re-configuring of the program itself.

#### Output trigger generation

The Boolean result of the evaluation was transformed into the TTL high or low level. To achieve this, we used a combination of FastEventServer (https://doi.org/10.5281/zenodo.3843624) and an Arduino-based single-board driver (https://doi.org/10.5281/zenodo.3515999) that enabled trigger generation at a latency of ∼100 μs (Sehara et al., 2019). Briefly, the server program received commands from the client program via the fast User Datagram Protocol (UDP), relayed them to the Arduino-based board via the USB1.1 cable, and finally responded back to the client program in UDP. The output TTL signal was connected to a LED positioned inside the camera’s field of view.

#### Data acquisition steps

During acquisition, the timestamps, frames, estimated whisker positions, and the status of the TTL output were first stored in memory, then once acquisition was finished these data were written to disk as a zip file containing serialized NumPy arrays (van der Walt et al., 2011). Two types of timestamps were collected: the frame-start timestamp, and the processing-end timestamp. The frame-start timestamp corresponds to the one obtained before triggering acquisition of each video frame. The processing-end timestamp occurred once the frame had been acquired and an output trigger had been generated.

### DNN models

We trained DNN models to estimate tips of three arc whiskers with the aid of the DeepLabCut 2.1.3 toolbox (Nath et al., 2019). One model was trained specifically for each mouse. As a base network of the DNN, the default ResNet-50 was used. The labeled whisker tips varied from one animal to another, depending on the length of the animal’s whiskers at the beginning of the course of behavioral sessions. We used the B2, C1, and β; the C2, C1, and β; or the B1, C1, and β whiskers to train the DNN model. Each DNN model detected a consistent set of whisker tips throughout the whole period of behavioral recordings.

#### Training of the models

Before starting any real-time feedback experiments, we ran a behavioral session to acquire videos for offline training of the DNN model. A set of 30- to 60-s videos at 100-Hz frame rate was acquired from each head-fixed mouse whisking freely without any constraints or task. The mini-batch K-means clustering method was used to extract 60–120 video frames in total from the set of videos to be used as the training dataset. Training of the DNN models typically required a total of one to three training sessions, each comprising ∼1,000,000 iterations. After each training session, 30 outlier frames were picked up from each video and added to the training data, using the default “jump” method provided in DeepLabCut 2.1 (Nath et al., 2019).

### Data analysis

Python (RRID:SCR_008394; version 3.7.7; van Rossum, 1995) was used to run *post hoc* analysis. In addition to the Python DeepLabCut toolbox, the following libraries were used during the analysis and annotation: NumPy (RRID:SCR_008633; version 1.19.1; van der Walt et al., 2011), SciPy (RRID:SCR_008058; version 1.5.2), matplotlib (RRID:SCR_008624; version 3.0.3; Hunter, 2007), pandas (RRID:SCR_018214; version 1.0.4; McKinney, 2010), scikit-image (version 0.17.2; van der Walt et al., 2014), Neo (RRID:SCR_000634; version 0.9.0; Garcia et al., 2014), h5py (version 2.10.0; Collette, 2013), and Jupyter notebook (RRID:SCR_013984). For *post hoc* annotation of videos, the pillow image processing library (version 7.1.2; https://python-pillow.org/) and the Python bindings of the OpenCV library (RRID:SCR_015526; opencv-python, version 3.4.9.33; https://opencv.org/) were used.

#### Latency profiling

During real-time experiments with the target interframe intervals of 10 ms, (1) the strobe-trigger digital output from the camera and (2) the pose-trigger output from the Arduino-based board were recorded at 10 kHz per channel using the Power1401 interface (CED) and Spike2 (RRID:SCR_000903). The resulting Spike2 files (27 sessions in total from three animals) were used to compute interframe intervals and the pose-trigger latency. For calculation of the time spent for internal procedures of our Python program, we recorded system timestamps using the Python “time” module. To profile the latency of image acquisition, timestamps were obtained before and after the Python function call to acquire a frame, and their differences were calculated. For estimation of the time spent for position estimation using DeepLabCut, the net duration including OpenCV-based down-sampling and DeepLabCut method calls (i.e., the total time spent to process the frame and obtain estimated positions) were measured. To profile latency for trigger generation, timestamps were collected before and after one transaction of commands, i.e., from the point when the client Python library dispatched the UDP packet to the server, to the point when the server program responded back to the UDP packet after the Arduino-based output board generated the trigger. For experiments when we varied the target interframe intervals from 10 to 20 ms, the resulting intervals were computed based on the frame-acquisition timestamps of the acquired videos.

#### Profiling of estimation accuracy

For each animal, 60 additional frames (20 from each 30-s video) were extracted from the video and were manually labeled independently of the training data using ImageJ (RRID:SCR_003070; https://imagej.nih.gov/). After training of the corresponding DNN model, the frames were subsampled and fed to the model. The resulting whisker positions were compared with the positions of manual labeling to compute error figures data from three animals were pooled and summarized together.

#### Profiling of real-time trigger accuracy

For each animal, we trained a distinct set of DNN models that perform *post hoc* estimation of the “true” positions of whisker tips in videos of real-time trigger generation. Based on this ground-truth data, kernel-density estimation was performed to generate event-density distributions of the position-values being evaluated in real time (i.e., the position of the middle whisker tip for the position-based evaluation, and the difference between the positions of the two whisker tips for the spread-based evaluation). A Gaussian distribution with a SD of 0.5 mm was used as the density kernel. To compute the conditional probability of trigger generation at each position, the density distribution during the triggered time points was divided by the distribution of the whole period of acquisition. The cumulative probability distribution of a Gaussian distribution was then fitted to the conditional probability distribution to estimate the position and variability of trigger threshold. The mean of the fitted Gaussian distribution was defined as the detected threshold position (note that closer to the set value is a better estimate), whereas the 2× SD value was considered to be the variability of the threshold (note that smaller values are more accurate).

To prevent the trigger accuracy figures from varying solely based on position-estimation accuracy, we used videos that had the mean per-frame part-wise estimation error of below 2 mm (whisker position-based triggering, *N* = 15 videos from six behavioral sessions from five animals; whisker spread-based triggering, *N* = 8 videos from four behavioral sessions from three animals).

#### Code and data availability

The code and the software described in our paper are freely available online (Pose-Trigger, https://doi.org/10.5281/zenodo.4459345; dlclib, https://doi.org/10.5281/zenodo.4459239; timedcapture, https://doi.org/10.5281/zenodo.4459208), and their source packages are available online at Python Package Index (PyPI, https://pypi.org/). The raw video data, the DeepLabCut models and the analytical procedures used in this study are available freely online (https://doi.org/10.12751/g-node.lgu2m0) and will be available on request.

## Results

### Real-time trigger generation based on whisker positions

Mice were head-fixed under the infrared illumination while high-speed video frames were acquired from the camera from above (Fig. 1*A*). To monitor the timing of the TTL output, a LED positioned in the field of view of the camera (Fig. 1*A*, inset, arrow) was set to flash in response to each trigger, allowing simultaneous capture of whisker positions in the video data and timing of the trigger signal. Our proof-of-concept experiment consisted of three phases: (1) acquisition of training video data for the DNN model; (2) training of the DNN model using DeepLabCut; and (3) applying the DNN model to real-time body-part estimation. Each training session for the DNN model took ∼12 h.

We set up our acquisition program so that whisker position estimation and trigger output generation were complete before the beginning of the acquisition of the next video frame (Fig. 1*B*). With the use of a busy-wait algorithm for triggering the acquisition of the next frame, actual interframe intervals varied in the range of 10–20 ms (Fig. 1*C*, left, median and 5% confidence intervals are shown for each condition). For the target interframe intervals of 10 ms, the resulting intervals were 11.27 ± 2.34 ms (mean ± SD, median 10.62 ms), or 91.02 ± 11.41 Hz. The variability in the interframe interval could be reduced by setting target intervals to 18 ms or larger. The resulting intervals then were 18.06 ± 0.79 ms (55.43 ± 1.48 Hz, mean ± SD, median 18.00 ms). These results were consistent with the profile of per-frame total processing time (Fig. 1*C*, right) which was 11.34 ± 2.27 ms (mean ± SD, grand average of *N* = 18,206 frames collected across sessions with different target interval settings).

To demonstrate the real-time flexibility of our program, we trained the DNN model to estimate tips of three whiskers in an arc, and used different evaluation algorithms for generating an output during a single behavioral session (Fig. 1*D*,*E*; Movie 1). In one configuration, the program was set to detect the position of a particular whisker, so that the LED switched on when the tip of the middle whisker was protracted across an arbitrary border (Fig. 1*D*). In another configuration, the distance between the two adjacent caudal whiskers was tracked, and the LED was only activated when the distance between the two whiskers was greater than an arbitrary threshold value (Fig. 1*E*; Movie 2). In both cases, the LED turned on one frame after the suprathreshold value was detected.

Movie 1. Example annotated video in a single representative session, when the position-based evaluation rule was applied. Annotation was performed *post hoc* based on the acquired data. Dots indicate the positions of three whiskers on one side of the mouse’s face. Flashes of the LED in the field of view (top left) reported the generation of output triggers in real time when the middle whisker (orange) protracted across the arbitrary border (dotted lines). The color of the border indicates the status of trigger output (white: off, orange: on). 10.1523/ENEURO.0415-20.2021.video.1

Movie 2. Example annotated video in a single representative session, when the distance-based evaluation rule was applied. The video was acquired during the same imaging session as in Video 1 and was annotated *post hoc*. The trigger was generated when the horizontal distance (arrow) between the two whiskers (cyan, green) went above the arbitrary threshold. The color of the arrow indicates the status of trigger output (white: off, orange: on). During the time of acquisition, the position estimation of the rostral-most tip (cyan) was unstable. Annotation was turned off when the tip was too caudal, too far behind the caudal-most tip (green). 10.1523/ENEURO.0415-20.2021.video.2

To measure the interframe intervals and feedback-trigger output latency more directly, we separately recorded the timing of the strobe-trigger output from the camera, as well as the timing of the feedback-trigger output at 10 kHz (Fig. 1*F*; Table 1). Our analyses revealed that, when the interframe interval was targeted at 10 ms, the actual interframe intervals we achieved occurred at ∼10.9 ms (91.7 Hz). The feedback-trigger output latency was ∼10.5 ms.

**Table 1:** Statistics of latencies

(per-video mean ± std, ms)	Mean	Std	Min	2.5%	Median	97.5%	Max
Frame intervals	10.90 ± 0.18	1.78 ± 0.37	9.77 ± 0.05	9.91 ± 0.04	10.35 ± 0.10	16.38 ± 1.63	26.95 ± 1.67
On-event latency	10.50 ± 0.26	1.70 ± 0.47	8.38 ± 2.94	9.48 ± 0.05	9.97 ± 0.16	15.44 ± 1.97	20.67 ± 3.41
Off-event latency	10.50 ± 0.29	1.69 ± 0.49	8.35 ± 2.97	9.46 ± 0.61	9.97 ± 0.21	15.03 ± 2.08	21.22 ± 3.27

The acquisition interval between frames, on-event latency, and off-event latency were summarized across multiple video acquisitions. For each parameter, the mean, SDs (Std), minimum values (Min), 2.5th percentiles (2.5%), median, 97.5th percentiles (97.5%), and the maximum values (Max) were averaged. *N* = 26 videos from six behavioral sessions taken from three animals (out of 27 videos in total, one video was excluded because there were less than 30 on/off events during the acquisition). Means and SDs are shown in milliseconds.

### Accuracy of real-time trigger generation

**Figure 2.**
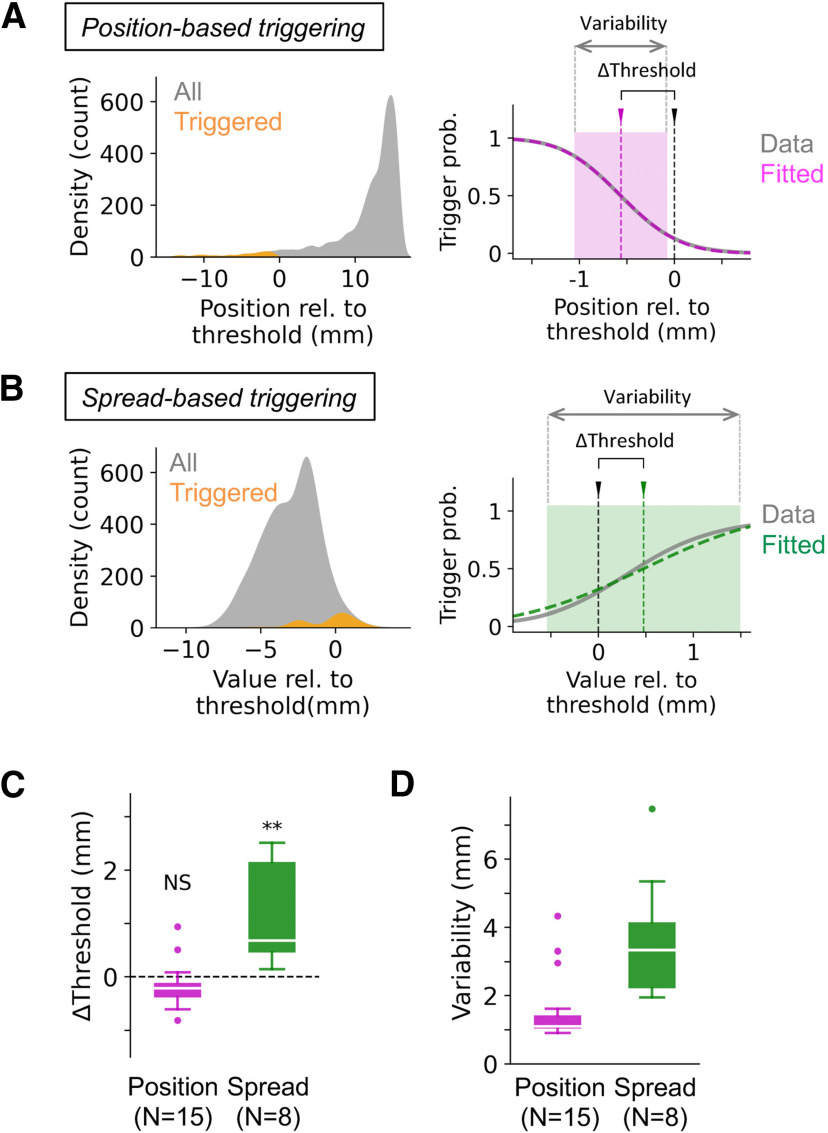
Accuracy of real-time trigger generation. ***A***, ***B***, Estimation of the accuracy of triggers based on video data. Density of occurrence, i.e., the number of frames, was estimated for the whole acquisition period of a video (left panels, gray) and for the period when the trigger was on (left panels, orange). The conditional probability of trigger generation was computed based on the two density distributions (right panels, gray). A Gaussian distribution (right panels, magenta (***A***) or green (***B***)) was fitted to the conditional probability distribution to estimate the difference between the set value and the actual threshold position (ΔThreshold) and the variability in the occurrence of the triggers (variability, double-headed arrows). The actual and estimated thresholds could vary by 1 mm. The density/counts in ***A*** are from a representative video where position-based triggers were generated, whereas the density/counts in ***B*** are from another representative video where triggers were generated based on the distance between whiskers. ***C***, ***D***, Summary of all position-based and spread-based acquisitions. The box plots are in the style of Tukey. For both conditions, the difference between the actual threshold position and the set value (***C***) was <1 mm on average, although the detected threshold value was significantly different from the value being set during acquisition (position-based, *p* = 0.0637, NS; spread-based, **p = 0.0078; Wilcoxon signed-rank test). Variability of trigger generation (***D***) was 1–3 mm on average. Compared with the whisker position-based trigger generation, the whisker spread-based triggering was less accurate. *N* = 15 videos (six behavioral sessions from five animals) for position-based trigger generation, and *N* = 8 videos (four behavioral sessions from three animals) for spread-based trigger generation.

To examine how accurately triggers were generated in real time, we trained a distinct set of DNN models that perform *post hoc* estimation of the true positions of whisker tips in videos of real-time trigger generation. Using this ground-truth data, event-frequency histograms were generated to estimate the conditional probability of trigger generation at each whisker position (Fig. 2*A*,*B*). Accuracy of real-time trigger generation could be then estimated as the mean and SD of the cumulative Gaussian distribution being fitted to the conditional probability distribution.

For both whisker position-based and whisker spread-based real-time trigger generations, the detected threshold value had a median accuracy of less than ±1 mm (Fig. 2*C*; Table 2). The position-based trigger generation was less variable (Fig. 2*D*, left; Table 2; median 1.10 mm, mean ± SD 1.59 ± 1.05 mm, *N* = 15 videos) than the whisker spread-based triggering which had a variance of ∼3 mm (Fig. 2*D*, right; Table 2; median 3.34 mm, mean ± SD 3.69 ± 1.90 mm, *N* = 8 videos). Both values of variance were well below the total variance in the values being evaluated in real time. The spread-based trigger generation also had higher threshold positions and was more variable compared with the position-based trigger generation. These differences could be related to the fact that the spread-based condition involved the estimation of the positions of two whisker tips, thus doubling the positional error compared with the evaluation based on the position of a single whisker.

**Table 2:** Per-video accuracy of real-time trigger generation

(values are in mm)		Mean	Std	Min	25%	Median	75%	Max
ΔThreshold	Position-based	−0.18	0.44	−0.81	−0.39	−0.21	−0.11	0.94
	Spread-based	1.10	0.91	0.15	0.47	0.63	1.85	2.44
Variability	Position-based	1.59	1.05	0.90	1.03	1.10	1.41	4.34
	Spread-based	3.69	1.90	1.95	2.21	3.34	4.15	7.50

In the top row (ΔThreshold), the average difference between the actual threshold and the value being set during acquisition for each video is shown. The bottom rows (variability) show the average variability in the threshold in individual videos. *N* = 15 videos (six behavioral sessions from five animals) for position-based trigger generation, and *N* = 8 videos (four behavioral sessions from three animals) for spread-based trigger generation.

### Latency profiling of DeepLabCut-based real-time trigger generation

**Figure 3.**
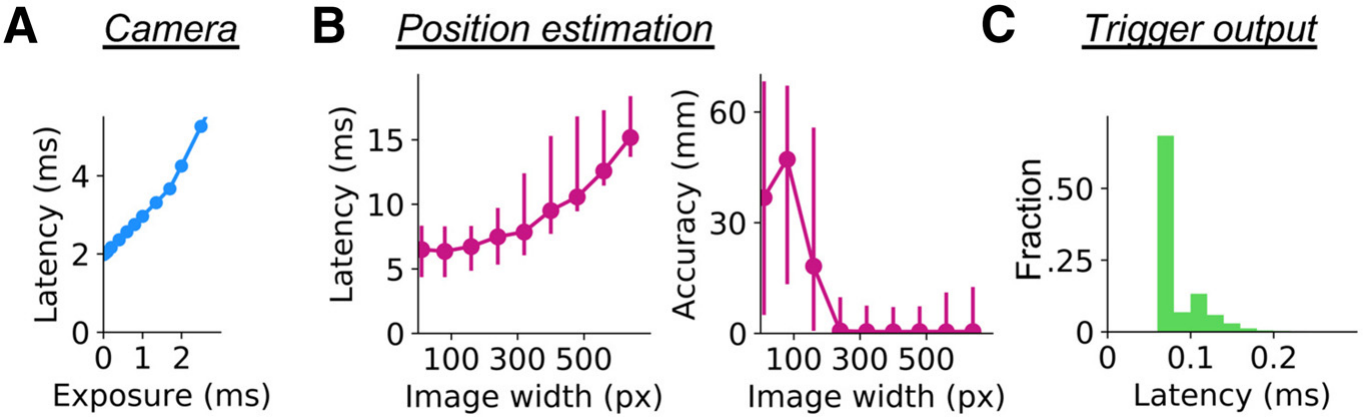
Profiling real-time acquisition procedures. ***A***, The latency to frame acquisition (*y*-axis) for different exposure settings was calculated. Median values from 2000 frames (640 × 480 pixels in size) per exposure setting are plotted. The results were so stable that 5% confidence intervals are not visible in the plot. ***B***, Latency (left) and accuracy (right) of DeepLabCut-mediated position estimation, using our model of whisker-position estimation from 180 frames acquired from three animals for each estimation condition. The latency estimation was made with both OpenCV-based subsampling and DeepLabCut-based body-part estimation. Dots represent the median values, and error bars stand for 5% confidence intervals. ***C***, Histogram of latency to output trigger generation. The output was turned on and off repeatedly for 3000 times, and the time spent for the Python call was measured each time.

To understand the processes that contribute to the ∼10.5-ms latency for generating an output, and to examine whether elements of the system could be sped up, we measured the time spent in each step separately during frame acquisition, whisker position estimation, and trigger generation (Fig. 3). Acquisition of a video frame, with the size of 640 × 480 pixels, took 2.36 ± 0.02 ms (mean ± SD, median 2.36 ms) for our default configuration of 400 μs exposure. When we lowered the exposure down to 5 μs, it still took 1.97 ± 0.09 ms (mean ± SD, median 1.98 ms) to acquire a single frame (Fig. 3*A*). The lower bound of ∼2 ms presumably corresponds to the time spent in transferring the frame data from the camera to the host computer. Our results are comparable to the theoretical minimum latency of 0.98 ms when a 16-bit image with the size of 640 × 480 pixels is transferred through USB3.1 communication (which has a theoretical maximum of 5 Gbps for generation 1).

For whisker position estimation, we measured the latency and accuracy at different settings of frame sizes (Fig. 3*B*). Here, the latency measure included the time spent for frame subsampling and position estimation. We subsampled the frame to the width of 320 pixels during our animal imaging experiments. The latency for this configuration was 8.08 ± 1.44 ms (mean ± SD, median 7.86 ms). Even when we used only 10 pixel-wide frames to pass on to the DNN model, the latency was 6.43 ± 1.03 ms (mean ± SD, median 6.49 ms; Fig. 3*B*, left). On the other hand, the accuracy of the estimation was significantly worse when frame size was reduced to less than half the original size (Fig. 3*B*, right).

Output triggers took only 0.10 ± 0.04 ms (mean ± SD, median 0.08 ms, *N* = 3000 frames; Fig. 3*C*). This latency was negligible compared with that of the acquisition and estimation steps. Together, under the conditions we used here, we conclude that the current trigger-output latency of 10.5 ms and the current interframe interval of 11 ms is close to the limit that our setup can achieve. The results of our profiling imply that there are bottlenecks in the steps of data transfer between different devices.

## Discussion

Here, we implemented DeepLabCut-mediated real-time feedback generation based on whisker positions. Our system can work at 80–90 Hz and can reliably generate an output in 10.5 ms. These values are within behavioral time scales (Bittner et al., 2017; Isett et al., 2018). This work highlights the fact that real-time feedback generation systems face a trade-off between speed and accuracy. Body-part estimation can be sped up by subsampling video frames, without any large degradation in accuracy. This strategy is likely to be suited to DNN model-based position estimation approaches including DeepLabCut because of their robust estimation based on noisy images.

### Challenges toward real-time multi-whisker detection

In the last 20 years, multiple research groups have reported increasingly sophisticated, easy-to-use tools for automated annotation of video data of animal behavior (Knutsen et al., 2005; Voigts et al., 2008; Perkon et al., 2011; Clack et al., 2012; Ohayon et al., 2013; Giovannucci et al., 2018; Dominiak et al., 2019; Vanzella et al., 2019; Betting et al., 2020; Petersen et al., 2020). One natural extension of this ability has been to apply these algorithms for on-line, closed-loop paradigms, where changes in behavior of the animal are detected as rapidly as possible, and the behavior is used to modify or manipulate the brain, the virtual environment or the context of behavior. From this perspective, the rodent whisker system, which has been a model system for understanding brain circuits related to sensory perception, movement and plasticity (Sachdev et al., 2001; Feldman and Brecht, 2005; Brecht et al., 2006; Diamond et al., 2008; Hooks, 2017), poses some unique challenges. Mice and rats have ∼30 similarly shaped whiskers on each side of the face, and they can sweep them back and forth at 10–25 Hz.

Alternatives to video-based real-time tracking do exist to overcome these challenges. For example, it is possible to use EMG for tracking the movement of the whiskers and whisker pad (Kleinfeld et al., 2002; Sachdev et al., 2003). It is also possible to attach a reflective marker to a whisker and optoelectronically track whisker movements reliably at high speeds (Bermejo et al., 1998). But approaches using EMG can be invasive and not targeted to individual whiskers. Tracking with reflective markers can be clumsy and limited to single whiskers. More recently, it has become possible to use neuromorphic approaches to achieve rapid (<3 ms) tracking of whiskers (Sehara et al., 2019). Another method has used a color-tracking camera to track multiple whiskers (Nashaat et al., 2017), with a latency of ∼30 ms, which is two to three times slower than the ∼10-ms behavioral time scale (Bittner et al., 2017; Isett et al., 2018). In addition, this method requires placing UV paint to whiskers every day. Although some of these earlier methods can achieve closed-loop latencies in tracking whiskers that are in behavioral time scales, these methods are either invasive or require markers, or are non-trivial to apply for real-time tracking of multiple appendages (i.e., whiskers) moving independently and rapidly.

DeepLabCut and other DNN-based marker-less approaches have a significant advantage over almost all earlier methods for offline pose estimation, and offline body-part tracking. Our current work extends the use of these approaches for real-time marker-less tracking of multiple whiskers. A few recent studies have made forays into real-time tracking with DeepLabCut (Forys et al., 2020; Kane et al., 2020). The first study using this approach demonstrated control of mouse behavior by rewarding particular forelimb movements, achieving a real-time tracking latency of 50 ms (Forys et al., 2020). The other one, using a different approach, has achieved a latency of 10 ms (Kane et al., 2020). But to achieve a 10-ms latency, Kane and colleagues subsampled each frame and restricted the resolution of video frames to ∼150 pixels in diameter. While this approach can work for tracking of limbs, it is not likely to be effective for tracking individual whiskers (compare Fig. 3*B*). In addition, it is not clear whether the video frames used for tracking are in fact being saved to disk for additional analysis.

One challenge that real-time tracking with video data poses is the computational cost of tracking objects from video frames. Fortunately, the power of processors and algorithms has increased and this has enabled processing of individual frames in <10 ms (Knutsen et al., 2005; Voigts et al., 2008; Perkon et al., 2011; Clack et al., 2012; Ohayon et al., 2013; Cao et al., 2018; Dominiak et al., 2019; Betting et al., 2020; Petersen et al., 2020). By comparison, little progress has been made to enhance acquisition latency of frames, i.e., the time required to transfer video frames between devices. As we document here, it can take milliseconds just to obtain a video frame (compare Fig. 3). Optimization of hardware/software interaction will be necessary to develop faster real-time tracking algorithms.

Here, by optimizing the acquisition and tracking procedures, we have built a system that is capable of closed-loop trigger generation in real time at 10.5-ms latency. The DNN-based approach affords the possibility of tracking body parts consistently across behavioral sessions without using any artificial markers. Triggers can be generated based on whisker position or on any other features of facial expression that are relevant for inferring the internal states of rodents (Dominiak et al., 2019; Stringer et al., 2019; Dolensek et al., 2020). In addition, our system implements the posture-evaluation mechanism that is easy to edit or update on-line during experiments. The approach we have developed here provides a generic real-time solution for any researcher in the field of behavioral neurophysiology.

### Bottlenecks of DNN-based tracking approaches

Profiling of our system revealed the major bottlenecks in the DNN-based approaches (Fig. 3). One major issue is the communication between devices: between the camera and the computer, and between the CPU memory and the GPU memory. The time required for body-part estimation using an image has a lower bound of around 6 ms, regardless of the number of pixels used. Considering the efficiency of the GPU during computation of the DNN model, and the fact that DeepLabCut runs faster in the batch mode (Mathis and Warren, 2018), it is reasonable to suspect that the transfer of the data to the graphics card is limiting the speed during this process.

Similar issues exist in the image acquisition and transfer from the camera to the host computer. Unlike frame-less real-time feedback approaches which can generate trigger outputs in ∼2 ms (Sehara et al., 2019), frame-based approaches take milliseconds just to obtain an image from the camera. While most open-loop video applications minimize the data transfer latency by allocating a buffer for incoming video frames, this strategy is not suitable for closed-loop applications that aim to minimize the latency from acquisition of an image to its processing.

There are several additional procedures for reducing the latency to output trigger generation using image-processing based real-time feedback systems. One effective procedure would be the use of more compact DNN models such as MobileNetV2 (Mathis et al., 2019). It could help reduce the load on the GPU and thus could lead to higher efficiency in body-part estimation (Kane et al., 2020). Restricting the region of interest in video frames could also help keep accuracy from degrading while speeding up the estimation process (Nath et al., 2019; Sehara et al., 2019).

It would be also beneficial to use a predictive model to compute future positions of the body parts (Kane et al., 2020). Assuming a certain restricted dynamics in the pattern of movement, predictions could work to detect a stereotypic pattern of movements one frame before it takes place (Kane et al., 2020). But the predictions might not be applicable for fast motion with large degrees of freedom where the implicit assumptions of the model may not always apply.

DNN-based approaches in the field of neuroscience have been evolving rapidly. Based on the same approach we used here on pose estimation, one could well imagine a DNN-based real-time feedback-generation system for other modalities, such as facial expression, vocalization, or population neural activity. Methods like ours are likely to contribute to the interrogation of relationships between virtual worlds, behavior and neural activity.

## Synthesis

Reviewing Editor: Mark Laubach, American University Decisions are customarily a result of the Reviewing Editor and the peer reviewers coming together and discussing their recommendations until a consensus is reached. When revisions are invited, a fact-based synthesis statement explaining their decision and outlining what is needed to prepare a revision will be listed below.

One minor change to the text and a recommendation by the reviewers are the only remaining issues for this manuscript. Please revise as soon as possible and I will handle by editorial review. Thank you for submitting your work to eNeuro.

- The authors should revise their citation of Forys et al. 2020 to emphasize that the study demonstrated control of mouse behavior by rewarding particular forelimb movements.

- The reviewers recommend the authors contribute their model to the DLC zoo.

## Author Response

To the editors,

We appreciate the helpful comments, suggestions and criticisms of the reviewers. We have tried to address all their concerns in this resubmission.

Reviewer 1

The authors describe an extension of previous work showing the application of deeplabcut in a real-time setting. Work here tracks whiskers of head fixed mice and provides examples where specific movements of individual whiskers could trigger behavioral feedback. While the work is impressive for describing very short latency feedback and potentially an extension of two previous papers Kane et al Elife and Forys et al eNeuro, it does have issues with being an accessible tool.

I am having trouble examining the software that the authors use. They give a couple of links to zenodo but unfortunately I was unable to open anything using these links in various combinations, please provide corrected links and/or attach the files. From what I can tell the authors have not provided an open source tool in a usable format. It is not explicitly clear that the authors will be sharing the full methods approach.

We now use URLs for the objects on Zenodo, in place of the DOIs (lines 118-119).

Please do note that in the review process for this journal we need to maintain our anonymity, and that poses some issues. Nevertheless, the libraries that we have developed in the course of the study are attached as zip archives of the repositories:

- Pose-Trigger repository: Extended Data 1

- dlclib repository: Extended Data 2

- timedcapture repository: Extended Data 3

Upon acceptance, we will publish the repositories at GitHub (https://github.com/), and the documentation at ReadTheDocs (https://readthedocs.org/), which we attached as Extended Data 4.

In addition, we have prepared a data repository on G-node (https://gin.g-node.org/). It contains all the raw data (raw videos, DeepLabCut projects, Spike2 recordings) and the Jupyter notebook to reproduce the analysis. We are going to publish it as soon as the acceptance of the paper.

Other limitations include the paper being somewhat unclear about response latencies as when one examines the figures and can see the LED turning on it looks as though this happens at about 25 milliseconds not the mentioned 12 milliseconds. I think they should include some records of the actual flash latency for repeated trials and potentially use higher frame rates if they are indeed going to conclude that latencies are so fast. It would be difficult to do this using a camera frame rate which is roughly equal to the latency.

2

The authors should use actual measured LED flash latencies and not software time stamps for performance calculations.

We have fixed this. We now show the distribution of the latencies based on recordings at a higher sampling rate (methods on lines 164-169). We showed the results as Figure 1F and Table 1. We describe the results on lines 251-256. In addition, we updated the latency description on Figure 1D and 1E to avoid any misleading impressions.

In a revision I would expect the authors to provide links to acquisition code and analysis code so that this potentially could be repeated and benefit others. The text mentions requests can be made, but it is unlcear that is a fully functioning system with proper documentation for others to use.

We are going to publish the python libraries to Python Package Index (PyPI, https://pypi.org/) at the time of acceptance. For the time being, the libraries that we have developed in the course of the study are attached as zip archives of the repositories (Extended Data 1-3).

In addition, for the main repository (pose-trigger), we prepare documentation at ReadTheDocs (https://readthedocs.org/). Here we attach the zip archive of the HTML documentation (Extended Data 4). Please do note that in the review process for this journal we need to maintain our anonymity, and that poses some issues.

These other links did not work for me: To achieve this, we used a combination of 108 FastEventServer (DOI:10.5281/zenodo.3843624) and an Arduino-based singleboard 109 driver (DOI:10.5281/zenodo.3515999) that enabled trigger generation at a latency of 110 ~ 100 microseconds (Sehara et al., 2019).

We changed the citation style from DOIs to URLs (lines 118-119).

Reviewer 2

The authors propose a method to use deeplabcut to extract whisker position and then provide a ttl pulse for real-time manipulations.

This latter facet may be the more innovative side, although several approaches already exist. The manuscript in its current version falls below the bar, but improvements could be made to greatly improve the product.

3

As this is not my field, upon conclusion of my review I did a pubmed search for real time whisker tracking. In ~30 minutes, my eyes were opened. Perkon et al 2011 and the several subsequent papers that use it (Ma 2017, Rahmati 2014, Romano 2018, 2020) achieves greater temporal resolution, in freely moving mice, and of all whiskers.

This BIOTACT tool has been used many times and is freely available on github. **Ma demonstrates 2ms resolution with the same camera used here** There is no mention of it. Others like Petersen et al plos comp bio 2020 are also not mentioned. While an interesting project, the manuscript is reporting on a new type of horse carriage after the car has been invented.

We added citations to some of these papers (lines 35-38, 324-328). Please do note that, while this earlier work "could have" "potentially" been used for tracking in close loop mode, none of these earlier studies actually showed any proof-of-concept success in tracking in real time or in the closed-loop mode. We added a section in Discussion describing the challenges for the offline tracking algorithms to be used in real time, and how our system overcomes the issues (lines 328-386).

My comments fall into three categories -the first two of which are critical.

1 - Performance benchmarking is absent. There is mention of a hand-scored dataset, but I see nno hits/misses/false alarms nor mention of ground truth, etc. Low latency is not enough. Any sense of accuracy is missing, which is somewhat troubling. Instead, our only metric is the avg busy-wait delayed frame rate.

We added more thorough analysis on the accuracy of estimation and timings of trigger generation (Figure 2). To this end, a deep-neural network was specifically trained for each video from the real-time triggering experiment (methods on lines 191-211), and on-line estimation was compared with the optimized off-line analysis (lines 261-281).

2 - The tool seems to work? (assuming point 1 is taken care of), but this problem has already been solved several times. What is this paper's impact? Make it clear that this is not just another method. The authors cite several recent papers from the Sachdev lab that are quite related, but it is entirely unclear to me what 8ms improved resolution (Nashaat), the benefit over similarly priced neuromorphic systems (Sehara), or what the fact that "DNN's are flexible" buys the potential user. While DLC is claimed to be easy, it is certainly non-trivial - particularly when compared to PixyCam. Also, Dominiak, published late last year, appears to achieve similar results with ~5ms resolution and a typical Baisler camera, albeit without real time detection.

We added paragraphs in Discussion describing the advantages of DNN-based methods over the other machine-vision approaches (lines 338-386). In addition, we made more explicit about the difference in the use of the terms "resolution" and "latency" (lines 47-54, 307-309). More specifically, while a Basler camera is capable of acquiring frames at less than 5 ms resolution, it is not capable of generating real-time triggers in 5 ms latency based on object positions, i.e. within 5 ms after the frame is captured.

4

3 - Please review your text before submitting. I empathize with the authors if not native english speakers - english is a nasty language - but it helps to review the draft and round it out. Likewise, many important citations are missing and the manuscript doesn't have a publication polish, particularly when compared to the papers mentioned above that the authors so commonly compare their work with.

We fixed the issues as suggested (please also see the points below).

major:

1C is the most important of the paper: " how well does this work?". I do not understand the vertical bars or "target" in 1C-left absolute (across a threshold) and relative (proximity) whisker position are mentioned. I would like to see quantification of these results in each version.

We added a section in Results describing the accuracy of real-time trigger generation (Figure 2, Table2; lines 261-281; methods on lines 191-211), by comparing the realtime results with DeepLabCut-based offline analysis.

I also assume? this is combined across all 3 animals, but I have no idea about if this is the case. Only "a mouse" is mentioned.

We fixed the reference on line 220. We also gave additional details about the number of sessions and animals used for real-time accuracy profiling on lines 209-211.

A DNN trained once would ideally generalize across an unlimited number of animals not used in the training data, but we have no inclination of this. More important still, I am not sure if the same whisker is identified on each animal and every time. The authors themselves do not indicate which whisker it is. If I as a researcher am interested in inter-whisker spacing or a whisker crossing a threshold, I may want to The same whisker is identified in each animal, every time. The same set of whiskers were consistently labeled. We added this point on lines 137-142. false positives are not discussed(!) I'd expect this to be quite a point of pride for the authors. As a user of both, I know that DLC - if well trained - is far more robust and reliable than Pixy.

We added a section in Results describing the accuracy of real-time trigger generation (Figure 2, Table 2; lines 261-281; methods on lines 191-211), by comparing the realtime results with DeepLabCut-based offline analysis.

5

openPose - while most often used with humans, should be cited when referring to 'behavioral monitoring algorithms'. More importantly, JAABA, MOTR is missing - these constitute a vital pillar for supervised algorithm to rodent behavioral research.

We added citation to these offline tracking libraries (lines 35-38, 49-50, 324-328).

line 126 - not vital, but why were the exact whiskers not known? if probably, I'd move this information to methods and not mention it in paper, simply use "three whiskers"

We have clarified this point, on lines 137-142.is the 20ms or so reported that PixyCam provides that much worse than 11.3ms? in the example shown (1D), the frames are separated by 11.5ms and 12.8ms? I do not see how this detection, delayed by potentially less than 8ms (though likely more) makes a difference. I am open and eager to be proven wrong, but it has to be in the paper.

Perhaps part of the missing quantification of performance can compare pixy to dlc, showing this method to have a greatly reduced failure rate. A new tool must in some way supplant the old one.

We were not clear about the distinction between temporal resolution and closed-loop latency. The actual delay with Pixycam reported in multiple papers (Nashaat et al., 2017 eNeuro; Nashaat et al., 2016 J Neurophys) is on the order of 30 ms. We have clarified this point (lines 47-49, 346-349).

Similarly, I don't see any sort of rivalring the excelent job done with 2ms resolution of Sehara 2019. I don't particularly think that the camera used here not specialized? it is not something most people own, and the overall cost, including high quality GPU is in the same ballpark as a neuromorphic camera (DVXplorer Lite is 2200 Euro). Again, I'm eager to find out why I would choose this approach over an existing one.

We added a more detailed comparison in the discussion (lines 344-353). One of the main reasons to use DeepLabCut-mediated approaches over other systems is their capability of tracking multiple body parts that are evident in a video. In addition, the DeepLabCut approach can be used to analyze the same video post hoc in detail. The acquisition steps and setups are simpler with this approach, which is the main advantage of DeepLabCut-based real-time system over other systems that use specialized sensors or cameras.

"DNN-based image-processing approaches are more versatile, more flexible, and allows researchers to track multiple body parts simultaneously. " it is unclear to me how this general quality of DNN's benefits this method. How does flexibility make this emthod better than previous methods? How it makes this an advancement over the Sehara paper with which the authors are well familiar with. I could see a similar method being used for lick or reach detection, but there is no 6 evidence that it would work and it is not mentioned in the paper. Much like Nashaat etal., this demonstration could greatly increase the impact of this research. Alternatively, DLC works regardless of head fixation. This would be a fantastic tool if the flexibility were extended to a head free mouse - even if the arena was quite small. It's questions like this that DLC was designed to address, rather than a new way to do something old.

Said more simply: make it clear to your reader what the advance is. As is, it reads as "we found yet another way to do something that is already possible". What are the benefits of this approach over the others?

We have added some of the points made up in this rebuttal to the Discussion (lines 323-386).

246 258. You refer to many specifics, but without citation. Please attribute this work (the authors may be thinking of Giovannucci et al., 2018; Vanzella et al, 2019; Yttri and Dudman, 2016; Bermejo and Zeigler, 2000; or)

We thank the reviewer for these citations, we have added a paragraph citing papers of behavioral tracking (lines 35-38, 324-328, 339-342). Please note that the approach used by Yittri and Dudman measures the movement of a joystick, not the movement of the animal's arm in real time. We cite Giovannucci et al., Vanzella et al., and Bermejo et al. papers.

While Kane 2020 is mentioned, it is not made clear that DLC Live should? be able to do this task just as well. Again, I encourage the authors to prove me wrong - a head to head comparison would be excellent to see and very appropriate.

We added a more detailed comparison in the discussion (lines 354-386). The DLC Live approach of Kane and colleagues is a good one, but as we show here coarse sampling of whisker movement data (something that DLC Live uses to characterize latency) makes our DLC algorithm inaccurate (Figure 3). Another issue is that, while we can and do routinely save all our video frames, it is not clear that DLC Live does so, too. Finally, as far as we know, the DLC Live library itself does not seem to control the frameacquisition step, which can be the source of delay.

minor:

line 28. sophisticated. In contrast...

line 39 - please break down run on sentence into 2 setnences.

similarly line 41 needs rewriting for clarity and flow

line 45 - comma after cheap - and consider the term inexpensive in its place.

line 69 - grabbed ->captured

line 94 were ->was

line 120 figure 1de.. if both, pelase use comma.

7

line 176 an LED

line 256, please break apart run-on sentence.

line 265, subject verb agreement / clarity

We fixed these points in the manuscript.
